# Noise annoyance and cardiovascular disease risk: results from a 10-year follow-up study

**DOI:** 10.1038/s41598-024-56250-8

**Published:** 2024-03-07

**Authors:** Omar Hahad, Donya Gilan, Matthias Michal, Oliver Tüscher, Julian Chalabi, Alexander K. Schuster, Karsten Keller, Lukas Hobohm, Volker H. Schmitt, Jochem König, Karl J. Lackner, Philipp Wild, Jörn M. Schattenberg, Andreas Daiber, Thomas Münzel

**Affiliations:** 1 Department of Cardiology, Cardiology I, University Medical Center of the Johannes Gutenberg University Mainz, Langenbeckstraße 1, 55131 Mainz, Germany; 2 German Center for Cardiovascular Research (DZHK), Partner Site Rhine-Main, Mainz, Germany; 3 Leibniz Institute for Resilience Research (LIR), Mainz, Germany; 4 Department of Psychiatry and Psychotherapy, University Medical Center of the Johannes Gutenberg-University Mainz, Mainz, Germany; 5 Department of Psychosomatic Medicine and Psychotherapy, University Medical Center of the Johannes Gutenberg-University Mainz, Mainz, Germany; 6 Institute for Molecular Biology, Mainz, Germany; 7 Preventive Cardiology and Preventive Medicine, Department of Cardiology, University Medical Center of the Johannes Gutenberg-University Mainz, Mainz, Germany; 8 Department of Ophthalmology, University Medical Center of the Johannes Gutenberg-University Mainz, Mainz, Germany; 9 Center for Thrombosis and Hemostasis (CTH), University Medical Center Mainz (Johannes Gutenberg-University Mainz), Mainz, Germany; 10 Medical Clinic VII, Department of Sports Medicine, University Hospital Heidelberg, Heidelberg, Germany; 11 Institute of Medical Biostatistics, Epidemiology and Informatics, University Medical Center of the Johannes Gutenberg-University Mainz, Mainz, Germany; 12 Institute of Clinical Chemistry and Laboratory Medicine, University Medical Center of the Johannes Gutenberg-University Mainz, Mainz, Germany; 13 Metabolic Liver Research Program, University Medical Center of the Johannes Gutenberg-University Mainz, Mainz, Germany; 14 Department of Internal Medicine II, Saarland University Medical Center, Homburg, Germany

**Keywords:** Noise annoyance, Environmental risk factor, Cardiovascular disease, Epidemiology, Cardiology, Epidemiology

## Abstract

The relationship between noise annoyance and risk of cardiovascular disease (CVD) still needs to be fully elucidated. Thus, we examined the relationship between noise annoyance and CVD risk in a large population-based cohort study. Cross-sectional (N = 15,010, aged 35–74 years, baseline investigation period 2007–2012) and prospective data (5- and 10-year follow-up from 2012 to 2022) from the Gutenberg Health Study were used to examine the relationship between noise annoyance due to different sources and risk of prevalent and incident CVD comprising atrial fibrillation, coronary artery disease, myocardial infarction, stroke, chronic heart failure, peripheral artery disease, and venous thromboembolism. In cross-sectional analyses, noise annoyance was an independent risk factor for prevalent CVD, with the strongest associations seen for noise annoyance during sleep (e.g., neighborhood noise annoyance: odds ratio 1.20, 95% confidence interval 1.13–1.27, *p* < 0.0001). While in the 10-year follow-up, mostly positive associations (although not significant) between noise annoyance and incident CVD were observed, no indication of increased CVD risk was observed after 5 years of follow-up. Noise annoyance due to different sources was associated with prevalent CVD, whereas only weak associations with incident CVD were found. Further large-scale studies are needed to establish the relationship between noise annoyance and risk of CVD.

## Introduction

Environmental noise exposure has been consistently demonstrated to increase the risk of cardiovascular disease (CVD)^1,2^. However, specific efforts to include environmental noise exposure as a manifest cardiovascular risk factor in preventive and treatment guidelines for CVD are still limited, which stems from the complexity of establishing a clear cause-and-effect relationship. This is of particular importance since noise exposure was not only shown to be prospectively associated with various CVD phenotypes, including arterial hypertension^3^, ischemic heart disease^4^, myocardial infarction^5^, stroke^6^, and atrial fibrillation^7^ in the general population but also to affect patients with pre-existing cardiovascular conditions adversely^8^. To further strengthen efforts to recognize noise exposure as an official health risk factor or at least risk enhancer, the noise-health/disease relationship must be fully investigated.

An ongoing question in the noise-health/disease research remains whether exposure to higher levels of environmental noise (physical dimension) and noise annoyance, as characterized by the subjective noise-induced stress reaction, is a reliable predictor of disease, in particular, of CVD. While it is acknowledged that noise annoyance is among the most prominent consequences of noise exposure in addition to noise-induced sleep disturbances^9^, the reliability of noise annoyance as an independent risk factor associated with CVD is questioned in the research community^10^. This is surprising since in the noise reaction model introduced by Babisch, the so-called non-auditory pathway clearly states that noise annoyance (among others) suggestive of stress reactions is a prerequisite for the contribution of noise in the development of CVD^11,12^. Nevertheless, this question is difficult to answer as to date most studies only focus on noise exposure levels and the few available studies that included noise annoyance are mostly inconsistent. For example, the only conducted meta-analysis from Ndrepepa and Twardella on the relationship between noise annoyance from road traffic noise and CVD showed an increased risk of arterial hypertension and a positive but insignificant association with risk of ischemic heart disease^13^. In contrast, in a German cohort including 2552 subjects, there was no evidence of a significant association between noise annoyance and prevalent hypertension and blood pressure^14^. However, based on the Gutenberg Health Study (GHS), a large prospective and population-based cohort from Germany, our group showed that noise due to different sources is associated with prevalent atrial fibrillation^15^.

There might be various hypotheses concerning the relationship between noise annoyance and its influence on CVD risk. Most probably a multifactorial pathomechanism must be suggested. First, noise annoyance may be causally related to CVD risk. Second, noise annoyance may be correlatively related to the development or aggravation of CVD risk factors, as noise annoyance and noise exposure share common variance. Third, noise annoyance is not causally related to CVD risk. Fourth, noise annoyance is not related to CVD risk due to poor reliability (only point estimates) and invalid assessment of noise annoyance as a psychological construct.

With the present study, we aimed to further focus on the association between noise annoyance and CVD by using data from the GHS. The novelty of the present study lies in its examination of a large cohort from the general population, investigating the association between noise annoyance from various sources, including both traffic and non-traffic sources, during daytime as well as sleep. This research uniquely explores prevalent as well as incident CVD comprising two follow-up time points, encompassing a diverse range of CVD disease phenotypes, including atrial fibrillation, coronary artery disease, myocardial infarction, stroke, chronic heart failure, peripheral artery disease, and venous thromboembolism, all within the same cohort.

## Results

### Characteristics of the study sample

Table 1 outlines the baseline and incident characteristics of the study sample, categorized by the level of overall noise annoyance at baseline (N = 14,639). Higher levels of noise annoyance correlated with a greater proportion of females, younger age, lower socioeconomic status, and increased use of earplugs. Additionally, certain cardiovascular risk factors, including hypertension, current smoking, and obesity, displayed varying prevalence across noise annoyance levels. With respect to prevalent CVD, the” extremely” annoyed group had the highest prevalence at 32.6%, while the “not at all” group showed a lower prevalence at 25.4%. However, the incidence of CVD at 5-year and 10-year follow-ups did not exhibit consistent differences among the noise annoyance categories. In terms of medication use, there were minimal variations across noise annoyance groups. Source-specific noise annoyance during the day and sleep demonstrated distinct patterns. During the day, road traffic noise annoyance increased from 38.6% in the "not at all" category to 59.9% in the "extremely" annoyed category. Similarly, aircraft noise annoyance during the day rose from 62.3 to 83.9%, railway noise annoyance increased from 13.8 to 21.7%, and industrial noise annoyance grew from 10.0 to 25.2% across these categories. Neighborhood noise annoyance during the day also demonstrated a clear upward trend from 40.3 to 48.2%. In the sleep context, the trends were even more pronounced. For road traffic noise, the annoyance increased from 9.5 to 31.7%, while aircraft noise annoyance rose from 19.2 to 68.7%. Similarly, railway noise annoyance increased from 5.1 to 15.4%, industrial noise annoyance from 1.2 to 6.3%, and neighborhood noise annoyance from 12.6 to 31.1%.

**Table 1:** Baseline and incident characteristics of the study sample stratified by the level of overall noise annoyance at baseline (*N* = 14,639).

Variable	Not at all (n = 3024)	Slightly (n = 3895)	Moderately (n = 3654)	Strongly (n = 2536)	Extremely (n = 1530)	*P* value
Female sex—% (no.)	51.5 (1556)	45.6 (1778)	49.6 (1811)	49.5 (1255)	54.4 (832)	**0.021**
Age—years	56.3 (11.0)	53.8 (11.0)	55.1 (11.3)	54.5 (11.2)	54.9 (10.7)	**0.0021**
Socioeconomic status—score	12.18 (4.44)	13.47 (4.42)	12.91 (4.44)	13.04 (4.52)	12.84 (4.41)	**0.00027**
Use of earplugs—% (no.)	2.3 (69)	3.4 (134)	4.7 (171)	7.0 (177)	10.4 (159)	**< 0.0001**
Time at current residence—years	17.00 (8.00/31.00)	15.00 (7.00/28.00)	16.00 (8.00/30.00)	15.00 (7.21/29.00)	15.00 (7.00/28.00)	0.060
Night shift work—% (no.)	7.6 (222)	8.8 (329)	6.9 (243)	7.8 (191)	8.0 (119)	0.61
Cardiovascular risk factors—% (no.)						
Diabetes mellitus	11.0 (331)	7.7 (299)	9.1 (333)	8.9 (226)	8.6 (131)	0.067
Hypertension	51.9 (1,569)	47.4 (1,844)	50.7 (1,851)	48.8 (1,236)	47.3 (724)	**0.043**
Current smoking	21.8 (658)	19.2 (746)	18.6 (678)	17.6 (447)	21.2 (324)	**0.048**
Obesity	28.4 (858)	23.0 (896)	24.2 (884)	25.6 (649)	24.0 (366)	**0.028**
Dyslipidemia	35.4 (1,068)	33.0 (1,282)	34.9 (1,273)	33.9 (857)	34.9 (531)	0.95
Family history of myocardial infarction or stroke	23.7 (716)	20.2 (786)	22.1 (809)	22.0 (558)	22.5 (344)	0.78
Cardiovascular disease (CVD)—% (no.)						
Prevalent CVD	25.4 (762)	23.7 (919)	27.3 (990)	29.2 (738)	32.6 (496)	**< 0.0001**
Incident CVD at 5-year follow-up	5.9 (108)	4.6 (119)	4.3 (99)	5.1 (78)	5.1 (43)	0.36
Incident CVD at 10-year follow-up	8.7 (97)	7.7 (131)	7.7 (107)	8.1 (76)	8.0 (40)	0.72
Medication—% (no.)						
Antidiabetic medication (A10)	7.0 (208)	5.1 (196)	5.9 (212)	6.2 (157)	6.0 (91)	0.62
Antithrombotic agents (B01)	13.8 (412)	9.9 (381)	13.0 (471)	11.7 (295)	11.9 (180)	0.46
Antihypertensives (C02)	1.1 (33)	1.0 (37)	0.9 (32)	1.1 (28)	1.2 (18)	0.76
Diuretics (C03)	6.2 (184)	3.9 (150)	5.4 (194)	5.3 (134)	5.6 (85)	0.85
Beta-blockers (C07)	17.9 (536)	15.5 (597)	16.8 (608)	16.7 (420)	17.4 (264)	0.93
Calcium channel blocker (C08)	8.2 (246)	6.1 (235)	7.1 (256)	7.4 (186)	7.7 (117)	1.00
Agents acting on the renin–angiotensin–aldosterone system (C09)	25.9 (775)	21.4 (823)	23.7 (859)	23.4 (589)	22.7 (345)	0.15
Lipid modifying agents (C10)	15.5 (462)	11.5 (442)	13.4 (485)	13.0 (326)	12.9 (196)	0.10
Source-specific noise annoyance (> 0)—% (no.)						
Road traffic noise annoyance (day)	–	38.6 (1,504)	55.1 (2,015)	63.1 (1,600)	59.9 (916)	**< 0.0001**
Aircraft noise annoyance (day)	–	62.3 (2,426)	75.3 (2,750)	82.2 (2,084)	83.9 (1,284)	** < 0.0001**
Railway noise annoyance (day)	–	13.8 (535)	19.1 (697)	22.1 (559)	21.7 (332)	**< 0.0001**
Industrial noise annoyance (day)	–	10.0 (390)	16.9 (618)	22.7 (574)	25.2 (385)	**< 0.0001**
Neighborhood noise annoyance (day)	–	40.3 (1,567)	46.2 (1,688)	50.1 (1,271)	48.2 (737)	** < 0.0001**
Road traffic noise annoyance (sleep)	–	9.5 (371)	20.5 (748)	31.1 (788)	31.7 (483)	**< 0.0001**
Aircraft noise annoyance (sleep)	–	19.2 (745)	37.4 (1,361)	57.0 (1,445)	68.7 (1,048)	**< 0.0001**
Railway noise annoyance (sleep)	–	5.1 (197)	10.3 (373)	14.8 (374)	15.4 (234)	**< 0.0001**
Industrial noise annoyance (sleep)	–	1.2 (48)	2.8 (102)	5.3 (135)	6.3 (96)	**< 0.0001**
Neighborhood noise annoyance (sleep)	–	12.6 (490)	20.1 (730)	26.8 (679)	31.1 (474)	**< 0.0001**

Continuous variables are shown as mean (standard deviation) or if skewness > 1 by median (Q1, Q3). Binary variables are described as relative and absolute frequencies. *P* values were calculated using the Jonckheere-Terpstra trend test. Socioeconomic status score ranges from 3 to 21 with higher values indicating higher status. Medication is labelled with the anatomical therapeutic chemical-code.

Significant values are in [bold].

### Association between source-specific noise annoyance and prevalent CVD

In cross-sectional analyses (Table 2), noise annoyance was associated with an increased risk of prevalent CVD after multivariable adjustment in all investigated noise sources (road traffic, aircraft, railway, industrial, and neighborhood). However, distinct sources of noise annoyance revealed differential impacts on prevalent CVD: while neighborhood noise annoyance was overall associated with a 15% (OR 1.15, 95% CI 1.11–1.20) elevated risk of prevalent CVD, the CVD risk from industrial noise annoyance was elevated by 12% (OR 1.12, 95% CI 1.06–1.19). Annoyance from road traffic and railway noise was accompanied by an 8% elevated risk of prevalent CVD (road traffic: OR 1.08, 95% CI 1.03–1.12; railway: OR 1.08, 95% CI 1.02–1.15).

**Table 2:** Cross-sectional association analysis between source-specific noise annoyance and prevalent CVD.

Noise annoyance	*N*	Model 3	
		OR per point increase [95% CI]	*P* value
During day			
Road traffic	13,784	1.05 [1.01; 1.10]	**0.017**
Aircraft	13,778	1.03 [0.99; 1.07]	0.095
Railway	13,766	1.05 [0.98; 1.12]	0.16
Industrial	13,769	1.11 [1.05; 1.18]	**0.00037**
Neighborhood	13,775	1.15 [1.10; 1.20]	**< 0.0001**
During Sleep			
Road traffic	13,747	1.15 [1.09; 1.22]	**< 0.0001**
Aircraft	13,742	1.08 [1.04; 1.12]	**< 0.0001**
Railway	13,735	1.15 [1.07; 1.24]	**0.00024**
Industrial	13,735	1.12 [0.97; 1.27]	0.11
Neighborhood	13,744	1.20 [1.13; 1.27]	**< 0.0001**
Overall			
Road traffic	13,784	1.08 [1.03; 1.12]	**0.00037**
Aircraft	13,781	1.04 [1.01; 1.08]	**0.010**
Railway	13,772	1.08 [1.02; 1.15]	**0.010**
Industrial	13,774	1.12 [1.06; 1.19]	**0.00015**
Neighborhood	13,775	1.15 [1.11; 1.20]	**< 0.0001**

Odds ratios (OR) and 95% confidence intervals (CI) are derived from a logistic regression model modeling for prevalent cardiovascular disease (CVD, composite variable comprising atrial fibrillation, coronary artery disease, myocardial infarction, stroke, chronic heart failure, peripheral artery disease, and venous thromboembolism per point increase in noise annoyance).

*N* denotes model 3.

Model 3 was adjusted for sex, age, socioeconomic status, night shift work, use of earplugs, years lived in residence, diabetes mellitus, arterial hypertension, smoking, obesity, dyslipidemia, family history of myocardial infarction or stroke, and medication use (diabetic drugs, antithrombotic agents, antihypertensives, diuretics, beta-blockers, calcium channel blocker, agents acting on the renin–angiotensin–aldosterone system, and lipid modifying agents).

Significant values are in [bold].

The risk from noise annoyance on prevalent CVD considerably differed between annoyance during the day and sleep, with a higher risk for annoyance during sleep: The risk from neighborhood noise annoyance during sleep was 1.3-fold higher compared to noise annoyance during the day, with an additional risk elevation of 5% (risk of prevalent CVD due to noise annoyance during the day: 15%, OR 1.15, 95% CI 1.10–1.20 vs. risk of prevalent CVD due to noise annoyance during sleep: 20%, OR 1.20, 95% CI 1.13–1.27). The risk from road traffic noise annoyance during sleep was even tripled compared to annoyance during sleep (5% risk elevation during day versus 15% risk elevation during sleep. Importantly, aircraft noise as well as railway noise annoyance during sleep was associated with an 8% respectively 15% risk elevation of prevalent CVD. Interestingly, industrial noise annoyance was solely identified as an independent risk factor during day after multivariable adjustment, with a risk elevation of 11%.

### Association between source-specific noise annoyance and incident CVD at 5- and 10-year follow-ups

At 5-year follow-up (Table 3), source-specific noise annoyance was mostly inconsistently and inversely associated (although not significant) with risk of incident CVD. Industrial noise annoyance was independently and inversely associated with risk of incident CVD (OR 0.34, 95% CI 0.09–0.74).

**Table 3:** Prospective association analysis between source-specific noise annoyance and incident CVD at 5-year follow-up.

Noise annoyance	*N*	Model 3	
		OR per point increase [95% CI]	*P* value
During day			
Road traffic	8589	0.93 [0.82; 1.03]	0.14
Aircraft	8585	0.98 [0.90; 1.07]	0.68
Railway	8581	0.91 [0.75; 1.08]	0.30
Industrial	8579	0.97 [0.81; 1.14]	0.74
Neighborhood	8585	1.02 [0.90; 1.15]	0.80
During Sleep			
Road traffic	8573	0.84 [0.69; 1.00]	0.064
Aircraft	8573	0.92 [0.83; 1.02]	0.11
Railway	8570	1.03 [0.83; 1.25]	0.76
Industrial	8568	0.34 [0.09; 0.74]	**0.034**
Neighborhood	8573	0.95 [0.80; 1.11]	0.52
Overall			
Road traffic	8589	0.91 [0.81; 1.01]	0.080
Aircraft	8587	0.98 [0.90; 1.06]	0.62
Railway	8583	0.94 [0.79; 1.10]	0.45
Industrial	8584	0.95 [0.79; 1.11]	0.53
Neighborhood	8585	1.02 [0.91; 1.14]	0.73

Odds ratios (OR) and 95% confidence intervals (CI) are derived from a logistic regression model modeling for incident cardiovascular disease (CVD, composite variable comprising atrial fibrillation, coronary artery disease, myocardial infarction, stroke, chronic heart failure, peripheral artery disease, and venous thromboembolism per point increase in noise annoyance).

*N* denotes model 3.

Model 3 was adjusted for sex, age, socioeconomic status, night shift work, use of earplugs, years lived in residence, diabetes mellitus, arterial hypertension, smoking, obesity, dyslipidemia, family history of myocardial infarction or stroke, and medication use (diabetic drugs, antithrombotic agents, antihypertensives, diuretics, beta-blockers, calcium channel blocker, agents acting on the renin–angiotensin–aldosterone system, and lipid modifying agents).

Significant values are in [bold].

At 10-year follow-up (Table 4), source-specific noise annoyance was mostly positively associated with increased risk of incident CVD, although not significant (e.g., industrial noise annoyance during the day: OR 1.11, 95% CI 0.94–1.29).

**Table 4:** Prospective association analysis between source-specific noise annoyance and incident CVD at 10-year follow-up.

Noise annoyance	*N*	Model 3	
		OR per point increase [95% CI]	*P* value
During day			
Road traffic	5335	1.01 [0.90; 1.13]	0.85
Aircraft	5333	1.05 [0.96; 1.14]	0.28
Railway	5326	1.10 [0.93; 1.28]	0.25
Industrial	5324	1.11 [0.94; 1.29]	0.18
Neighborhood	5330	1.00 [0.88; 1.13]	0.97
During Sleep			
Road traffic	5324	1.05 [0.89; 1.22]	0.55
Aircraft	5325	1.05 [0.96; 1.15]	0.28
Railway	5322	1.10 [0.90; 1.33]	0.32
Industrial	5321	1.01 [0.66; 1.41]	0.98
Neighborhood	5324	0.92 [0.76; 1.09]	0.35
Overall			
Road traffic	5335	1.00 [0.90; 1.12]	0.94
Aircraft	5334	1.05 [0.96; 1.14]	0.27
Railway	5328	1.08 [0.92; 1.25]	0.31
Industrial	5329	1.10 [0.94; 1.28]	0.22
Neighborhood	5330	0.96 [0.85; 1.08]	0.52

Odds ratios (OR) and 95% confidence intervals (CI) are derived from a logistic regression model modeling for incident cardiovascular disease (CVD, composite variable comprising atrial fibrillation, coronary artery disease, myocardial infarction, stroke, chronic heart failure, peripheral artery disease, and venous thromboembolism per point increase in noise annoyance).

*N* denotes model 3.

Model 3 was adjusted for sex, age, socioeconomic status, night shift work, use of earplugs, years lived in residence, diabetes mellitus, arterial hypertension, smoking, obesity, dyslipidemia, family history of myocardial infarction or stroke, and medication use (diabetic drugs, antithrombotic agents, antihypertensives, diuretics, beta-blockers, calcium channel blocker, agents acting on the renin–angiotensin–aldosterone system, and lipid modifying agents).

### Association between overall noise annoyance and prevalent as well as incident CVD at 5- and 10-year follow-ups

Table 5 displays the results of the association between overall noise annoyance and prevalent as well as incident CVD at 5- and 10-year follow-ups. Consistently, overall noise annoyance was associated with risk of prevalent CVD, with higher effect estimates observed for overall noise annoyance during sleep: While overall noise annoyance was associated with an elevated CVD risk of 12% (OR 1.12, 95% CI 1.08–1.15), overall daytime noise annoyance was associated with a 9% (OR 1.09, 95% CI 1.05–1.12) increased risk of CVD and noise annoyance during sleep was accompanied with a 13% (OR 1.13, 95% CI 1.10–1.17) risk elevation. Regarding risk of incident CVD, no associations were observed for overall noise annoyance at 5- and 10-year follow-ups.

**Table 5:** Cross-sectional/prospective association analysis between overall noise annoyance and prevalent/incident CVD.

Overall noise annoyance	N	Model 3	
		OR per point increase [95% CI]	*P* value
Prevalent CVD			
Overall noise annoyance	13,785	1.12 [1.08; 1.15]	**< 0.0001**
Overall noise annoyance day	13,785	1.09 [1.05; 1.12]	**< 0.0001**
Overall noise annoyance sleep	13,752	1.13 [1.10; 1.17]	**< 0.0001**
Incident CVD at 5-year follow-up			
Overall noise annoyance	8590	0.99 [0.92; 1.07]	0.84
Overall noise annoyance day	8590	0.99 [0.91; 1.07]	0.75
Overall noise annoyance sleep	8576	0.94 [0.86; 1.03]	0.19
Incident CVD at 10-year follow-up			
Overall noise annoyance	5336	1.01 [0.93; 1.10]	0.86
Overall noise annoyance day	5336	1.01 [0.93; 1.10]	0.79
Overall noise annoyance sleep	5336	1.02 [0.93; 1.11]	0.68

Odds ratios (OR) and 95% confidence intervals (CI) are derived from a logistic regression model modeling for prevalent/incident cardiovascular disease (CVD, composite variable comprising atrial fibrillation, coronary artery disease, myocardial infarction, stroke, chronic heart failure, peripheral artery disease, and venous thromboembolism per point increase in noise annoyance).

*N* denotes model 3.

Model 3 was adjusted for sex, age, socioeconomic status, night shift work, use of earplugs, years lived in residence, diabetes mellitus, arterial hypertension, smoking, obesity, dyslipidemia, family history of myocardial infarction or stroke, and medication use (diabetic drugs, antithrombotic agents, antihypertensives, diuretics, beta-blockers, calcium channel blocker, agents acting on the renin–angiotensin–aldosterone system, and lipid modifying agents).

Significant values are in [bold].

## Discussion

To our knowledge, this is the first study that comprehensively and prospectively examined the association between multiple sources of noise annoyance, including the differentiation between exposure during the day and sleep and the risk of prevalent and incident CVD comprising atrial fibrillation, coronary artery disease, myocardial infarction, stroke, chronic heart failure, peripheral artery disease, and venous thromboembolism in a large population-based cohort. The results of the present study demonstrate an independent association between noise annoyance and prevalent CVD. The present study revealed a differing impact of various noise sources on the risk of prevalent CVD elucidating the relevance of noise sources that people are exposed to regarding the risk of CVD. Furthermore, the results of the present study highlighted the importance of timing in which noise annoyance occurs and thereby elucidated the significant burden of noise annoyance during sleep on prevalent CVD.

Chronic stress due to noise exposure was shown to induce oxidative stress, promote endothelial dysfunction, and activate inflammatory and prothrombotic pathways^16,17^. By this, a neurobiological pathway that includes the stress-induced activity of the amygdala and increased arterial inflammation was supposed as a central mechanism in the induction of noise-induced CVD including death due to myocardial infarction, myocardial infarction, heart failure, and coronary and peripheral revascularization^18^. This is likely due to the circumstance that noise annoyance causes an increase in blood pressure, stress hormone levels, endothelial dysfunction, oxidative stress, arterial stiffness, and vascular inflammation^16,19–22^. Fortunately, the negative effects on blood pressure and arterial stiffness were shown to be reversible in the case of noise exposure and accompanying annoyance reduction^23^. Beyond, the present study revealed a differing impact on CVD risk in response to various sources of noise annoyance. Interestingly, the CVD risk from neighborhood and industrial noise annoyance was higher than road traffic and railway noise annoyance. These data must be further investigated in future studies. The heightened association between neighborhood noise annoyance and CVD may stem from the continuous and diverse nature of neighborhood noise, incorporating various sources and stressors. Chronic exposure coupled with potential psychosocial stressors related to the living environment may contribute to a more sustained impact on cardiovascular health.

The present study compared the risk of CVD due to noise annoyance during the day and sleep. The risk from overall noise annoyance during sleep was higher than during the daytime. Also, regarding the specific investigated noise sources, the risk of prevalent CVD was higher during sleep and slightly less harmful during the daytime. In the present study, the CVD risk from road traffic and railway noise annoyance during sleep was tripled compared to annoyance during the day, and neighborhood noise annoyance during the sleep was associated with an increased CVD risk of 33% compared to daytime annoyance. Overall, neighborhood noise was the most harmful source of annoyance regarding CVD risk in the present investigation and as might have been expected, industrial noise annoyance was not significantly associated with CVD during sleep/night-hours, potentially caused by lower noise levels due to government regulations in the nighttime. In contrast, industrial noise annoyance during daytime was significantly associated with prevalent CVD. Sleep is a vital neurophysiological state with reduced sympathetic tone and increased parasympathetic tone, resulting in a lower heart rate and blood pressure linked with a cardio-protective effect^24^. Nighttime noise is associated with stress hormone increase, vascular oxidative stress, endothelial dysfunction, increased vascular stiffness, hypertension, and inflammation^22^. Simulated nighttime aircraft noise caused adrenaline release and led to impairment of the endothelial function partly due to oxidative stress in healthy adults and a more pronounced vascular dysfunction in subjects with already established CVD^25^. Notably, the negative vascular effects of nighttime aircraft noise were found to be independent from annoyance and attitude towards noise^8^. This may contribute to short sleep duration, fragmented sleep, reduced slow-wave sleep, psychological stress, and insomnia^24^. These sleep disturbances might foster cardio-metabolic pathways that adversely affect cardiovascular health and therefore, studies revealed that nighttime noise with disrupted or disturbed sleep as a risk factor for CVD^8,24,26^. Aircraft noise as punctually very loud short-term load with increased stress levels was also particularly harmful in the present study during sleep-time, which was in line with literature^24,26^.

In the present study, noise annoyance was not significantly associated with incident CVD in previously CVD-naïve participants. This finding implicates some hypotheses: firstly, it seems probable, that noise annoyance and the development of incident CVD follow a dose–response relationship, and a long-term chronic dose is needed for CVD development. The differences in risk observed between the 5-year and 10-year follow-up analyses, particularly in source-specific assessments, may indicate evolving associations over time. Variations in sample sizes and incident events, potentially impacting statistical power, could contribute to the lack of significant associations over the course of the study. Secondly, it has to be assumed that different types of stress responders to noise annoyance exist. In this context, noise annoyance was shown to differ between different characteristics and personality traits and was supposed to underly an individual variability^27^. Thirdly, although it is plausible that noise annoyance may induce stress, pre-existing stress could also enhance sensitivity to noise, leading to increased noise annoyance. Within this framework, stress emerges as a broader underlying factor influencing both noise annoyance and CVD over time, making it a more accurate predictor^28^.

The strength of the present study lies in the large cohort of the general population (> 15,000 individuals) with the highly standardized anamnestic and clinical investigation, including detailed follow-up. Since this study was performed in Germany, a study limitation is that the extrapolation of the findings to other ethnicities or countries must be done with caution, as well as to cohorts with varying age ranges. The study’s observational, partly cross-sectional nature does not allow for causal inferences, and residual confounding cannot be entirely excluded. As we had no data concerning objective noise exposure indicators, we considered noise annoyance to be a valid indicator of adverse noise-induced effects. It is essential to recognize that noise annoyance, in our study, may be influenced by factors beyond the measured exposure levels. This could include individual sensitivities, psychological factors, or even contextual aspects of the living environment. Individuals who are more susceptible to noise exposure may reside in quieter areas, yet still experience annoyance at lower noise levels. This aspect highlights the complex interplay between subjective responses to noise and the actual levels of exposure. We further did not assess whether participants have moved during the follow-up period, representing a potential source of misclassification, which may have interfered with the present results.

In conclusion, annoyance from traffic, railway, aircraft industrial, and neighborhood noise was associated with an elevated risk of prevalent but not incident CVD. These risks were pronounced when noise annoyance occurred during sleep. The present study supports the call for strategies to reduce environmental noise exposure and thus noise annoyance, in particular during the nighttime.

## Methods

### Study design and sample

The GHS is an observational, single-center cohort study from Mid-Western Germany. Comprehensive information on study design and further details were published previously^29–31^. Briefly, 15,010 individuals aged 35–74 years underwent a standardized 5-h-lasting baseline-examination performed from 2007 to 2012 at the study center at the University Medical Center Mainz, Germany. These examinations included various interviews and clinical examinations conducted in compliance with standard operating procedures. The follow-up examinations took place in 5-year periods, i.e., from 2012 to 2017 (follow-up 1) and from 2017 to 2022 (follow-up 2).

Additionally, an extensive telephone interview was conducted between the follow-up cycles at the study center, thus 2.5 years and 7.5 years after the baseline examination. All procedures conducted in the GHS were approved by the ethics committee of the Statutory Physician Board of the State Rhineland-Palatinate [reference number 837.020.07(5555)] and the local data safety commissioners and were in line with the ethical principles for medical research involving human subjects as outlined in the Declaration of Helsinki. Before the inclusion of participants, written informed consent was obtained.

### Noise annoyance

Self-reported noise annoyance was measured in standardized and validated fashion, as reported recently^15,32^. Based on a 5-point Likert scale ranging from “not at all”, over “slightly”, “moderately”, and “strongly” to “extremely”, subjects were asked to rate “how annoyed have you been in the past years by … during the day/in your sleep?”. Multiple sources of annoyance were assessed, including road traffic, aircraft, railway, industrial, and neighborhood noise (noise from the surrounding apartment environment and within the building in multi-family residences). Overall noise annoyance was defined as the highest annoyance rating regardless of the specific noise source and whether it affected daytime or sleep. Likewise, source-specific overall noise annoyance was defined as the highest source-specific annoyance rating regardless of whether it affected daytime or sleep. We employed noise annoyance at baseline as a predictor, given its relatively stable nature across various follow-up time points, as depicted in Supplementary Figs. S1 and S2.

### Prevalent and incident cardiovascular disease

Prevalent and incident CVD were assessed based on medical records (physician diagnosis) or diagnosis during study visit and were defined as the presence of any of the following diseases: atrial fibrillation, coronary artery disease, myocardial infarction, stroke, chronic heart failure, peripheral artery disease, and venous thromboembolism.

### Definition of covariates

Information concerning sociodemographic variables, cardiovascular risk factors, and medication intake from the 5-h baseline examination were used to provide a comprehensive statistical adjustment strategy. Detailed definitions of the covariates used in the present study be found in^15,32^.

### Statistical analysis

The characteristics of the study sample are presented based on the level of overall noise annoyance at baseline, with mean and standard deviation for continuous variables. If skewness is greater than 1, median (Q1, Q3) is reported for continuous variables. Binary variables are described in terms of relative and absolute frequencies. To assess statistical trends across levels of overall noise annoyance, the Jonckheere-Terpstra trend test was employed. Logistic regression analyses with corresponding odds ratios (OR), 95% confidence intervals (CI), and *p* values were used to determine the relationship between noise annoyance and prevalent and incident CVD (composite variable comprising atrial fibrillation, coronary artery disease, myocardial infarction, stroke, chronic heart failure, peripheral artery disease, and venous thromboembolism). Noise annoyance was considered a continuous variable in all models. Consequently, OR can be interpreted for each point increase in noise annoyance. The incident analysis was only conducted in those subjects without CVD at baseline. Incident analyses were conducted at 5- as well as 10-year follow-ups (thus analyzing 5-year as well as 10-year cumulative incidence). Statistical analysis included sequential adjustment: Model 1 was adjusted for sex (binary) and age (continuous). Model 2 was additionally adjusted for socioeconomic status (continuous), use of earplugs (binary), years lived in residence (continuous), and night shift work (binary). Model 3 was additionally adjusted for diabetes mellitus (binary), arterial hypertension (binary), current smoking (binary), obesity (binary), dyslipidemia (binary), family history of MI or stroke (binary), and medication use (diabetic drugs, antithrombotic agents, antihypertensives, diuretics, beta-blockers, calcium channel blocker, agents acting on the renin–angiotensin–aldosterone system, and lipid modifying agents, all binary). For clarity, we have included only the results for model 3 in the main manuscript. Detailed results on sequential adjustments (model 1–3) can be found in the Online Supplementary Tables S1 to S4. In the present analysis, *p* values should be treated as a continuous measure of the statistical strength of an association, and they are therefore reported exactly. For descriptive reasons, *p* values < 0.05 were regarded as significant associations. The statistical data analyses were performed using the software R (http://www.r-project.org/).

### Supplementary Information

Supplementary Information.

## Data Availability

The analysis presents clinical data of a large-scale population-based cohort with ongoing follow-up examinations. This project constitutes a major scientific effort with high methodological standards and detailed guidelines for analysis and publication to ensure scientific analyses on the highest level. Therefore, data are not made available for the scientific community outside the established and controlled workflows and algorithms. To meet the general idea of verification and reproducibility of scientific findings, we offer access to data at the local database in accordance with the ethics vote on request at any time. The GHS steering committee, which comprises a member of each involved department and the head of the GHS, convenes once a month. The steering committee decides on internal and external access of researchers and use of the data and biomaterials based on a research proposal to be supplied by the researcher. Interested researchers make their requests to the head of the GHS (Philipp S. Wild, philipp.wild@unimedizin-mainz.de).
