# Supplementary material for: Noise annoyance and cardiovascular disease risk: results from a 10-year follow-up study
Source: Sci Rep. 2024 Mar 7;14:5619. doi: 10.1038/s41598-024-56250-8 (PMC10920781; doi:10.1038/s41598-024-56250-8)
Supplement: Supplementary file 1 — Supplementary Information. [file 41598_2024_56250_MOESM1_ESM.docx]

**Noise annoyance and cardiovascular disease risk – results from a 10-year follow-up study**

**Online-Supplement**

Omar Hahad PhD^1,2*^, Donya Gilan^3,4^, Matthias Michal MD^5,2^, Oliver Tüscher MD^3,4,6^, Julian Chalabi B.Sc.^7^, Alexander K. Schuster MD^8^, Karsten Keller MD^1,9,10^, Lukas Hobohm MD^1,9^, Volker H. Schmitt MD^1,2^, Jochem König PhD^11^, Karl J. Lackner MD^12^, Philipp Wild MD^7,9,2,6^, Jörn M. Schattenberg MD^13,14^, Andreas Daiber PhD^1,2,9^, Thomas Münzel MD^1,2,9^

^1^ Department of Cardiology – Cardiology I, University Medical Center of the Johannes Gutenberg-University Mainz, Mainz, Germany

^2^ German Center for Cardiovascular Research (DZHK), partner site Rhine-Main, Mainz, Germany

^3^ Leibniz Institute for Resilience Research (LIR), Mainz, Germany

^4^ Department of Psychiatry and Psychotherapy, University Medical Center of the Johannes Gutenberg-University Mainz, Mainz, Germany

^5^ Department of Psychosomatic Medicine and Psychotherapy, University Medical Center of the Johannes Gutenberg-University Mainz, Mainz, Germany

^6^ Institute for Molecular Biology, Mainz, Germany

^7^ Preventive Cardiology and Preventive Medicine, Department of Cardiology, University Medical Center of the Johannes Gutenberg-University Mainz, Mainz, Germany

^8^ Department of Ophthalmology, University Medical Center of the Johannes Gutenberg-University Mainz, Mainz, Germany

^9^ Center for Thrombosis and Hemostasis (CTH), University Medical Center Mainz (Johannes Gutenberg-University Mainz), Mainz, Germany

^10^ Medical Clinic VII, Department of Sports Medicine, University Hospital Heidelberg, Heidelberg, Germany

^11^ Institute of Medical Biostatistics, Epidemiology & Informatics, University Medical Center of the Johannes Gutenberg-University Mainz, Mainz, Germany

^12^ Institute of Clinical Chemistry and Laboratory Medicine, University Medical Center of the Johannes Gutenberg-University Mainz, Mainz, Germany

^13^ Metabolic Liver Research Program, University Medical Center of the Johannes Gutenberg-University Mainz, Mainz, Germany

^14^ Department of Internal Medicine II, Saarland University Medical Center, Homburg

***Address for correspondence:**

Omar Hahad, PhD

Department of Cardiology, Cardiology I

University Medical Center of the Johannes Gutenberg University Mainz

Langenbeckstraße 1, 55131 Mainz, Germany

Email: [omar.hahad@unimedizin-mainz.de](mailto:omar.hahad@unimedizin-mainz.de)

Phone: +49 (0) 6131-17-2476

Fax: +49 (0) 6131-17-3420

**Figure S1.** Heatmaps displaying Pearson correlation coefficients (Corr) for various sources of noise annoyance at baseline, 5-year follow-up (FU), and 10-year follow-up (FU10). The left graphic represents continuous variables, while the right graphic represents binary variables (coded as <0).

**Figure S2.** Distribution of total noise annoyance at baseline (BL), 5-year follow-up (FU), and 10-year follow-up (FU10) displayed through boxplots.

**Table S1.** Cross-sectional association analysis between source-specific noise annoyance and prevalent CVD.

| Noise annoyance | *N* | Model 1 | | Model 2 | | Model 3 | |
| --- | --- | --- | --- | --- | --- | --- | --- |
|  |  | OR per point increase [95% CI] | *P* value | OR per point increase [95% CI] | *P* value | OR per point increase [95% CI] | *P* value |
| During day | | | | | | | |
| Road traffic | 13,784 | 1.06 [1.02; 1.10] | **0.0039** | 1.04 [1.00; 1.09] | **0.033** | 1.05 [1.01; 1.10] | **0.017** |
| Aircraft | 13,778 | 1.01 [0.98; 1.04] | 0.55 | 1.01 [0.98; 1.05] | 0.36 | 1.03 [0.99; 1.07] | 0.095 |
| Railway | 13,766 | 1.08 [1.02; 1.14] | **0.013** | 1.07 [1.00; 1.13] | **0.035** | 1.05 [0.98; 1.12] | 0.16 |
| Industrial | 13,769 | 1.13 [1.06; 1.19] | **<0.0001** | 1.11 [1.05; 1.18] | **0.00024** | 1.11 [1.05; 1.18] | **0.00037** |
| Neighborhood | 13,775 | 1.17 [1.12; 1.22] | **<0.0001** | 1.15 [1.10; 1.20] | **<0.0001** | 1.15 [1.10; 1.20] | **<0.0001** |
| During Sleep | | | | | | | |
| Road traffic | 13,747 | 1.15 [1.09; 1.21] | **<0.0001** | 1.13 [1.07; 1.19] | **<0.0001** | 1.15 [1.09; 1.22] | **<0.0001** |
| Aircraft | 13,742 | 1.06 [1.02; 1.10] | **0.00064** | 1.07 [1.03; 1.10] | **0.00020** | 1.08 [1.04; 1.12] | **<0.0001** |
| Railway | 13,735 | 1.17 [1.09; 1.25] | **<0.0001** | 1.15 [1.07; 1.23] | **0.00016** | 1.15 [1.07; 1.24] | **0.00024** |
| Industrial | 13,735 | 1.11 [0.98; 1.26] | 0.11 | 1.11 [0.98; 1.26] | 0.10 | 1.12 [0.97; 1.27] | 0.11 |
| Neighborhood | 13,744 | 1.21 [1.15; 1.27] | **<0.0001** | 1.19 [1.13; 1.26] | **<0.0001** | 1.20 [1.13; 1.27] | **<0.0001** |
| Overall | | | | | | | |
| Road traffic | 13,784 | 1.08 [1.04; 1.12] | **<0.0001** | 1.07 [1.03; 1.11] | **0.0012** | 1.08 [1.03; 1.12] | **0.00037** |
| Aircraft | 13,781 | 1.02 [0.99; 1.05] | 0.13 | 1.03 [1.00; 1.06] | 0.058 | 1.04 [1.01; 1.08] | **0.010** |
| Railway | 13,772 | 1.10 [1.05; 1.17] | **0.00027** | 1.09 [1.03; 1.15] | **0.0020** | 1.08 [1.02; 1.15] | **0.010** |
| Industrial | 13,774 | 1.13 [1.07; 1.19] | **<0.0001** | 1.12 [1.06; 1.18] | **0.00011** | 1.12 [1.06; 1.19] | **0.00015** |
| Neighborhood | 13,775 | 1.17 [1.13; 1.22] | **<0.0001** | 1.16 [1.11; 1.20] | **<0.0001** | 1.15 [1.11; 1.20] | **<0.0001** |

Odds ratios (OR) and 95% confidence intervals (CI) are derived from a logistic regression model modeling for prevalent cardiovascular disease (CVD, composite variable comprising atrial fibrillation, coronary artery disease, myocardial infarction, stroke, chronic heart failure, peripheral artery disease, and venous thromboembolism per point increase in noise annoyance)

*N* denotes model 3

Model 1 was adjusted for sex and age

Model 2 was additionally adjusted for socioeconomic status, night shift work, use of earplugs, and years lived in residence

Model 3 was additionally adjusted for diabetes mellitus, arterial hypertension, smoking, obesity, dyslipidemia, family history of myocardial infarction or stroke, and medication use (diabetic drugs, antithrombotic agents, antihypertensives, diuretics, beta-blockers, calcium channel blocker, agents acting on the renin-angiotensin-aldosterone system, and lipid modifying agents)

**Table S2.** Prospective association analysis between source-specific noise annoyance and incident CVD at 5-year follow-up.

| Noise annoyance | *N* | Model 1 | | Model 2 | | Model 3 | |
| --- | --- | --- | --- | --- | --- | --- | --- |
|  |  | OR per point increase [95% CI] | *P* value | OR per point increase [95% CI] | *P* value | OR per point increase [95% CI] | *P* value |
| During day | | | | | | | |
| Road traffic | 8,589 | 0.93 [0.83; 1.03] | 0.19 | 0.92 [0.82; 1.03] | 0.14 | 0.93 [0.82; 1.03] | 0.14 |
| Aircraft | 8,585 | 0.96 [0.88; 1.04] | 0.28 | 0.97 [0.89; 1.06] | 0.50 | 0.98 [0.90; 1.07] | 0.68 |
| Railway | 8,581 | 0.92 [0.77; 1.09] | 0.36 | 0.90 [0.75; 1.07] | 0.26 | 0.91 [0.75; 1.08] | 0.30 |
| Industrial | 8,579 | 0.99 [0.83; 1.16] | 0.91 | 0.99 [0.83; 1.16] | 0.87 | 0.97 [0.81; 1.14] | 0.74 |
| Neighborhood | 8,585 | 1.05 [0.93; 1.17] | 0.46 | 1.04 [0.92; 1.17] | 0.50 | 1.02 [0.90; 1.15] | 0.80 |
| During Sleep | | | | | | | |
| Road traffic | 8,573 | 0.87 [0.72; 1.02] | 0.11 | 0.84 [0.69; 1.00] | 0.068 | 0.84 [0.69; 1.00] | 0.064 |
| Aircraft | 8,573 | 0.90 [0.82; 0.99] | **0.038** | 0.91 [0.82; 1.00] | 0.068 | 0.92 [0.83; 1.02] | 0.11 |
| Railway | 8,570 | 1.03 [0.83; 1.24] | 0.76 | 1.03 [0.83; 1.24] | 0.80 | 1.03 [0.83; 1.25] | 0.76 |
| Industrial | 8,568 | 0.33 [0.09; 0.72] | **0.031** | 0.33 [0.09; 0.73] | **0.033** | 0.34 [0.09; 0.74] | **0.034** |
| Neighborhood | 8,573 | 0.96 [0.81; 1.12] | 0.60 | 0.96 [0.81; 1.13] | 0.67 | 0.95 [0.80; 1.11] | 0.52 |
| Overall | | | | | | | |
| Road traffic | 8,589 | 0.92 [0.82; 1.02] | 0.11 | 0.90 [0.80; 1.00] | 0.059 | 0.91 [0.81; 1.01] | 0.080 |
| Aircraft | 8,587 | 0.95 [0.88; 1.03] | 0.25 | 0.97 [0.89; 1.05] | 0.44 | 0.98 [0.90; 1.06] | 0.62 |
| Railway | 8,583 | 0.94 [0.80; 1.10] | 0.47 | 0.93 [0.78; 1.09] | 0.37 | 0.94 [0.79; 1.10] | 0.45 |
| Industrial | 8,584 | 0.96 [0.81; 1.13] | 0.65 | 0.96 [0.80; 1.12] | 0.62 | 0.95 [0.79; 1.11] | 0.53 |
| Neighborhood | 8,585 | 1.04 [0.93; 1.16] | 0.47 | 1.04 [0.93; 1.16] | 0.44 | 1.02 [0.91; 1.14] | 0.73 |

Odds ratios (OR) and 95% confidence intervals (CI) are derived from a logistic regression model modeling for incident cardiovascular disease (CVD, composite variable comprising atrial fibrillation, coronary artery disease, myocardial infarction, stroke, chronic heart failure, peripheral artery disease, and venous thromboembolism per point increase in noise annoyance)

*N* denotes model 3

Model 1 was adjusted for sex and age

Model 2 was additionally adjusted for socioeconomic status, night shift work, use of earplugs, and years lived in residence

Model 3 was additionally adjusted for diabetes mellitus, arterial hypertension, smoking, obesity, dyslipidemia, family history of myocardial infarction or stroke, and medication use (diabetic drugs, antithrombotic agents, antihypertensives, diuretics, beta-blockers, calcium channel blocker, agents acting on the renin-angiotensin-aldosterone system, and lipid modifying agents)

**Table S3.** Prospective association analysis between source-specific noise annoyance and incident CVD at 10-year follow-up.

| Noise annoyance | *N* | Model 1 | | Model 2 | | Model 3 | |
| --- | --- | --- | --- | --- | --- | --- | --- |
|  |  | OR per point increase [95% CI] | *P* value | OR per point increase [95% CI] | *P* value | OR per point increase [95% CI] | *P* value |
| During day | | | | | | | |
| Road traffic | 5,335 | 1.03 [0.92; 1.14] | 0.64 | 1.01 [0.90; 1.13] | 0.84 | 1.01 [0.90; 1.13] | 0.85 |
| Aircraft | 5,333 | 1.03 [0.95; 1.12] | 0.49 | 1.04 [0.96; 1.14] | 0.32 | 1.05 [0.96; 1.14] | 0.28 |
| Railway | 5,326 | 1.09 [0.93; 1.27] | 0.25 | 1.08 [0.92; 1.26] | 0.33 | 1.10 [0.93; 1.28] | 0.25 |
| Industrial | 5,324 | 1.14 [0.98; 1.32] | 0.085 | 1.12 [0.95; 1.30] | 0.14 | 1.11 [0.94; 1.29] | 0.18 |
| Neighborhood | 5,330 | 1.03 [0.90; 1.16] | 0.69 | 1.01 [0.89; 1.15] | 0.83 | 1.00 [0.88; 1.13] | 0.97 |
| During Sleep | | | | | | | |
| Road traffic | 5,324 | 1.07 [0.92; 1.24] | 0.37 | 1.05 [0.90; 1.22] | 0.51 | 1.05 [0.89; 1.22] | 0.55 |
| Aircraft | 5,325 | 1.04 [0.95; 1.13] | 0.42 | 1.05 [0.95; 1.14] | 0.33 | 1.05 [0.96; 1.15] | 0.28 |
| Railway | 5,322 | 1.11 [0.91; 1.33] | 0.27 | 1.09 [0.89; 1.31] | 0.39 | 1.10 [0.90; 1.33] | 0.32 |
| Industrial | 5,321 | 1.06 [0.72; 1.46] | 0.72 | 0.99 [0.65; 1.38] | 0.95 | 1.01 [0.66; 1.41] | 0.98 |
| Neighborhood | 5,324 | 0.94 [0.79; 1.11] | 0.51 | 0.93 [0.77; 1.10] | 0.42 | 0.92 [0.76; 1.09] | 0.35 |
| Overall | | | | | | | |
| Road traffic | 5,335 | 1.02 [0.92; 1.13] | 0.73 | 1.00 [0.90; 1.12] | 0.94 | 1.00 [0.90; 1.12] | 0.94 |
| Aircraft | 5,334 | 1.03 [0.95; 1.12] | 0.45 | 1.04 [0.96; 1.13] | 0.32 | 1.05 [0.96; 1.14] | 0.27 |
| Railway | 5,328 | 1.07 [0.92; 1.24] | 0.34 | 1.06 [0.91; 1.23] | 0.42 | 1.08 [0.92; 1.25] | 0.31 |
| Industrial | 5,329 | 1.13 [0.97; 1.30] | 0.098 | 1.11 [0.95; 1.28] | 0.19 | 1.10 [0.94; 1.28] | 0.22 |
| Neighborhood | 5,330 | 0.99 [0.88; 1.11] | 0.89 | 0.98 [0.87; 1.10] | 0.74 | 0.96 [0.85; 1.08] | 0.52 |

Odds ratios (OR) and 95% confidence intervals (CI) are derived from a logistic regression model modeling for incident cardiovascular disease (CVD, composite variable comprising atrial fibrillation, coronary artery disease, myocardial infarction, stroke, chronic heart failure, peripheral artery disease, and venous thromboembolism per point increase in noise annoyance)

*N* denotes model 3

Model 1 was adjusted for sex and age

Model 2 was additionally adjusted for socioeconomic status, night shift work, use of earplugs, and years lived in residence

Model 3 was additionally adjusted for diabetes mellitus, arterial hypertension, smoking, obesity, dyslipidemia, family history of myocardial infarction or stroke, and medication use (diabetic drugs, antithrombotic agents, antihypertensives, diuretics, beta-blockers, calcium channel blocker, agents acting on the renin-angiotensin-aldosterone system, and lipid modifying agents)

**Table S4.** Cross-sectional/prospective association analysis between overall noise annoyance and prevalent/incident CVD.

| Overall noise annoyance | *N* | Model 1 | | Model 2 | | Model 3 | |
| --- | --- | --- | --- | --- | --- | --- | --- |
|  |  | OR per point increase [95% CI] | *P* value | OR per point increase [95% CI] | *P* value | OR per point increase [95% CI] | *P* value |
| *Prevalent CVD* | | | | | | | |
| Overall noise annoyance | 13,785 | 1.11 [1.08; 1.15] | **<0.0001** | 1.11 [1.07; 1.14] | **<0.0001** | 1.12 [1.08; 1.15] | **<0.0001** |
| Overall noise annoyance day | 13,785 | 1.09 [1.05; 1.12] | **<0.0001** | 1.08 [1.04; 1.11] | **<0.0001** | 1.09 [1.05; 1.12] | **<0.0001** |
| Overall noise annoyance sleep | 13,752 | 1.12 [1.09; 1.15] | **<0.0001** | 1.12 [1.09; 1.15] | **<0.0001** | 1.13 [1.10; 1.17] | **<0.0001** |
| *Incident CVD at 5-year follow-up* | | | | | | | |
| Overall noise annoyance | 8,590 | 0.98 [0.90; 1.06] | 0.58 | 0.99 [0.91; 1.07] | 0.80 | 0.99 [0.92; 1.07] | 0.84 |
| Overall noise annoyance day | 8,590 | 0.97 [0.90; 1.05] | 0.50 | 0.98 [0.91; 1.07] | 0.71 | 0.99 [0.91; 1.07] | 0.75 |
| Overall noise annoyance sleep | 8,576 | 0.93 [0.85; 1.01] | 0.091 | 0.94 [0.86; 1.02] | 0.15 | 0.94 [0.86; 1.03] | 0.19 |
| *Incident CVD at 10-year follow-up* | | | | | | | |
| Overall noise annoyance | 5,336 | 1.01 [0.93; 1.09] | 0.90 | 1.01 [0.93; 1.10] | 0.84 | 1.01 [0.93; 1.10] | 0.86 |
| Overall noise annoyance day | 5,336 | 1.01 [0.93; 1.09] | 0.87 | 1.01 [0.93; 1.10] | 0.77 | 1.01 [0.93; 1.10] | 0.79 |
| Overall noise annoyance sleep | 5,336 | 1.01 [0.93; 1.09] | 0.84 | 1.01 [0.93; 1.10] | 0.74 | 1.02 [0.93; 1.11] | 0.68 |

Odds ratios (OR) and 95% confidence intervals (CI) are derived from a logistic regression model modeling for prevalent/incident cardiovascular disease (CVD, composite variable comprising atrial fibrillation, coronary artery disease, myocardial infarction, stroke, chronic heart failure, peripheral artery disease, and venous thromboembolism per point increase in noise annoyance)

*N* denotes model 3

Model 1 was adjusted for sex and age

Model 2 was additionally adjusted for socioeconomic status, night shift work, use of earplugs, and years lived in residence

Model 3 was additionally adjusted for diabetes mellitus, arterial hypertension, smoking, obesity, dyslipidemia, family history of myocardial infarction or stroke, and medication use (diabetic drugs, antithrombotic agents, antihypertensives, diuretics, beta-blockers, calcium channel blocker, agents acting on the renin-angiotensin-aldosterone system, and lipid modifying agents)
